# Social hierarchy shapes behavioral and transcriptional responses to chronic stress and ketamine in male mice

**DOI:** 10.1016/j.isci.2026.116825

**Published:** 2026-07-21

**Authors:** Serena Gasperoni, Xiuqi Ji, Choham Sudre-Chinsky, Eduardo Cáceres Pajuelo, Fatemeh Sadat Zolfaghari, Otto Boldemann, Manar Manla Hasan, Tommaso Biagini, Daniil Umanski, Yair Shemesh, Aron Kos, Paula Fontanet, Alon Chen, Juan Pablo Lopez

**Affiliations:** 1 Department of Neuroscience, Karolinska Institutet, 17165 Stockholm, Sweden; 2 Department of Molecular Neuroscience, Weizmann Institute of Science, Rehovot 7610001, Israel; 3 Department of Stress Neurobiology and Neurogenetics, Max Planck Institute of Psychiatry, Munich, Germany

**Keywords:** chronic social defeat stress, social hierarchy, ketamine, antidepressant response, automated behavioral profiling, social box, medial prefrontal cortex, mPFC, ventral hippocampus, vHipp, RNA sequencing

## Abstract

Chronic stress is a major risk factor for psychiatric disorders, yet the mechanisms underlying individual differences in vulnerability and treatment response remain poorly understood. Using the Social Box, a seminatural environment, we performed high-resolution, continuous tracking of group-housed male mice during 11 days, capturing behavior during both active and inactive phases. We found that social hierarchy within a group strongly shapes stress outcomes, with dominant individuals exhibiting amplified behavioral alterations following chronic stress, including rest fragmentation and disrupted social interactions. A single ketamine administration mitigated these effects, restoring baseline behavioral signatures in dominant individuals. Bulk mRNA sequencing was performed in the medial prefrontal cortex and ventral hippocampus. This revealed a hierarchy-dependent transcriptional response with 141 differentially expressed genes in the medial prefrontal cortex of dominant, ketamine-treated mice. Overall, this study provides a framework for examining stress and pharmacological interventions in group-housed mice, enabling high-resolution, longitudinal analyses under semi-naturalistic conditions.

## Highlights

• Stress-induced behavioral changes vary across the circadian cycle
• Dominant mice exhibit greater vulnerability to chronic stress
• A single dose of ketamine reverses stress-induced abnormalities in dominant mice
• Ketamine induces transcriptional changes in the mPFC of dominant mice

## Graphical abstract

**Figure:**
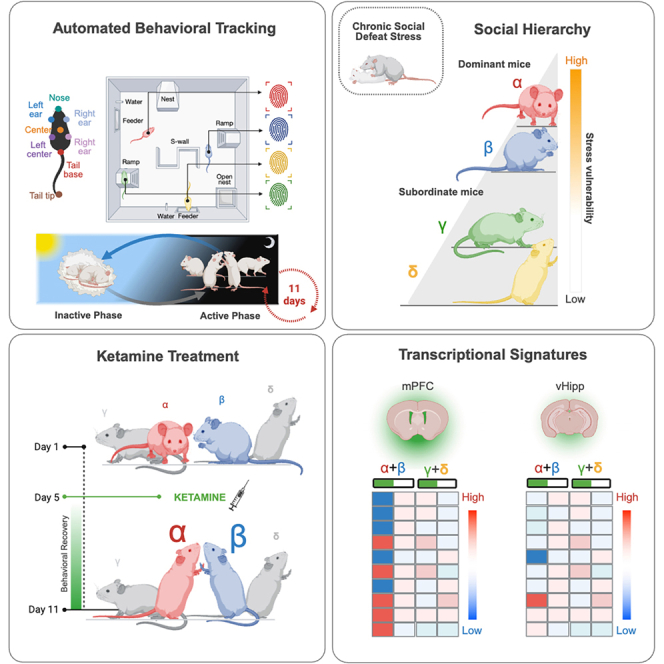

## Teaser

Natural sciences; Biological sciences; Neuroscience; Behavioral neuroscience; Molecular neuroscience

## Introduction

Chronic stress is a major risk factor in the development of a variety of severe and impairing psychiatric conditions, including major depressive disorder (MDD), post-traumatic stress disorder (PTSD), and anxiety disorders, among others.^1^^,^^2^^,^^3^ These conditions affect millions worldwide, and over 84 million people in Europe alone,^4^ imposing profound human and economic costs. Despite this, standard treatments such as selective serotonin reuptake inhibitors (SSRIs) remain constrained by modest efficacy, pronounced interindividual variability, and a delayed onset of therapeutic action,^5^ underscoring the urgent need for more effective interventions. Building on this clinical challenge, understanding the biological mechanisms and behavioral outcomes underlying stress vulnerability is essential for developing improved therapeutic strategies.

Despite the rising global incidence of stress-related disorders, the mechanisms driving maladaptive stress responses and individual variability remain poorly understood. In rodents, some of these features, or endophenotypes, can be modeled using chronic stress paradigms, such as the chronic social defeat stress (CSDS), which induces robust phenotypes including anhedonia, despair, and social withdrawal, among others.^6^ However, most traditional behavioral assessments rely on short, reductionist tests that capture only isolated behavioral components, overlooking individual differences and longitudinal dynamics and the influence of social behaviors within a group context.^7^ This limitation hampers our ability to fully characterize stress-induced phenotypes and their evolution over time. To address these limitations, it is critical to consider social dynamics that shape stress responses.

Social hierarchies represent a critical but often neglected factor in stress research. Across species, including rodents, non-human primates, and humans, dominance rank is a key determinant in shaping an individual’s behavior, its relationship with the environment and its conspecifics, as well as an individual's vulnerability to stress.^8^^,^^9^ Yet, studies examining the interaction between social rank and stress susceptibility have yielded inconsistent results. Some report increased vulnerability in subordinate individuals,^10^ while others suggest maladaptive responses in dominant animals,^11^^,^^12^^,^^13^ or no clear hierarchy effect at all.^14^ Disentangling these relationships requires approaches that capture both individual and group-level dynamics. Because rodents are nocturnal, it is also essential to monitor behavior across both active (dark) and inactive (light) phases to capture the full spectrum of stress-related phenotypes and avoid bias toward daytime activity. To address this gap, we employed the Social Box (SB),^15^ an enriched, semi-naturalistic environment that enables continuous, automated tracking of group-housed mice. This new method allows high-resolution, longitudinal, and individual behavioral profiling (i.e., hours, days, weeks), providing a unique opportunity to examine how chronic stress and social hierarchy interact over time.

While behavioral context is essential, treatment strategies remain a parallel priority. Over the past two decades, ketamine, an NMDA receptor antagonist, has gained substantial attention in the clinic as a rapid-acting antidepressant, revolutionizing the treatment landscape for mood disorders. S-ketamine, the enantiomer with the highest NMDA receptor binding affinity, received approval in 2019 by the Food and Drug Administration (FDA) and the European Medicines Agency (EMA) for clinical use in patients with difficult-to-treat depression (DTD) and acute suicidality.^16^ Unlike conventional antidepressants, ketamine produces rapid and, in some cases, sustained symptom relief.^17^ However, the mechanisms underlying its positive antidepressant effects have not been fully elucidated yet. In addition, it remains unclear why clinical response is highly variable, with some patients significantly benefiting from it, while others do not. Preclinical models offer a powerful means to investigate these mechanisms, enabling longitudinal behavioral characterization and the identification of factors (i.e., behavioral or biological) that could predict treatment response. Understanding these mechanisms requires linking behavioral outcomes to molecular changes in stress-sensitive brain regions.

Among them, the medial prefrontal cortex (mPFC) and ventral hippocampus (vHipp) have consistently emerged as key areas in circuits regulating emotion, cognition, and stress adaptation. Both clinical and preclinical studies report structural and molecular alterations in these regions following chronic stress, including neuronal atrophy, glial loss, and disrupted synaptic plasticity.^18^^,^^19^^,^^20^ For example, chronic stress reduces the expression of key receptors (e.g., AMPAR subunits) and proteins involved in synaptic plasticity (e.g., synapsin 1), impairing excitatory transmission and plasticity.^21^ Importantly, these same brain regions are implicated in ketamine’s antidepressant effects. By mediating increased glutamate signaling in the mPFC, ketamine has been suggested to promote synaptogenesis and synaptic plasticity.^22^^,^^23^ In the vHipp, NMDAR blockage by ketamine has been shown to promote the translation of proteins involved in synaptic plasticity and to be implicated in the transcriptional regulation of genes whose expression is altered by chronic stress.^24^^,^^25^^,^^26^ Yet, how social hierarchy and stress interact with ketamine and subsequently shape the transcriptional landscape of these regions remains largely unexplored. To bridge this gap, we combined high-throughput behavioral and molecular approaches in a unified experimental framework.

In this study, we performed an automated, high-resolution longitudinal analysis of the effects of chronic stress and a single ketamine administration in group-housed male mice over an 11-day period, spanning both active (dark) and inactive (light) phases. By continuously recording behavior across 24-h cycles, including both 12-h active and 12-h inactive phases, we captured temporal dynamics often overlooked in conventional stress paradigms. Using the SB, this approach revealed persistent stress-induced behavioral alterations, such as rest fragmentation and disrupted social interactions, that were amplified in dominant individuals within a group, highlighting a critical interplay between hierarchy and stress response. A single ketamine administration mitigated these effects, restoring both baseline behavioral signatures and specific stress-sensitive behaviors in dominant mice. In parallel, we applied bulk mRNA sequencing to generate a high-throughput molecular profile of the mPFC and vHipp, examining how chronic stress, social hierarchy, and ketamine interact at behavioral and transcriptional levels. Our analysis uncovered a hierarchy dependent, region-specific transcriptional response, where ketamine induced 141 differentially expressed genes (DEGs) in the mPFC of dominant mice. In addition, we observed enrichment in postsynaptic compartments, consistent with its known mechanistic targets. By integrating longitudinal analyses of complex, ethologically relevant behavioral data with region-specific transcriptomic profiling, we are the first to demonstrate that social rank critically shapes both behavioral and molecular responses to chronic stress and ketamine. This points to synapse-related molecular signatures as a potential mechanism underlying ketamine’s sustained antidepressant effects and establishes a foundation for long-term, high-resolution studies of stress and pharmacological interventions in semi-naturalistic environments.

## Results

### Longitudinal analysis of complex social behaviors in group-housed male mice identifies critical time windows of stress-related phenotypes

**Figure 1:**
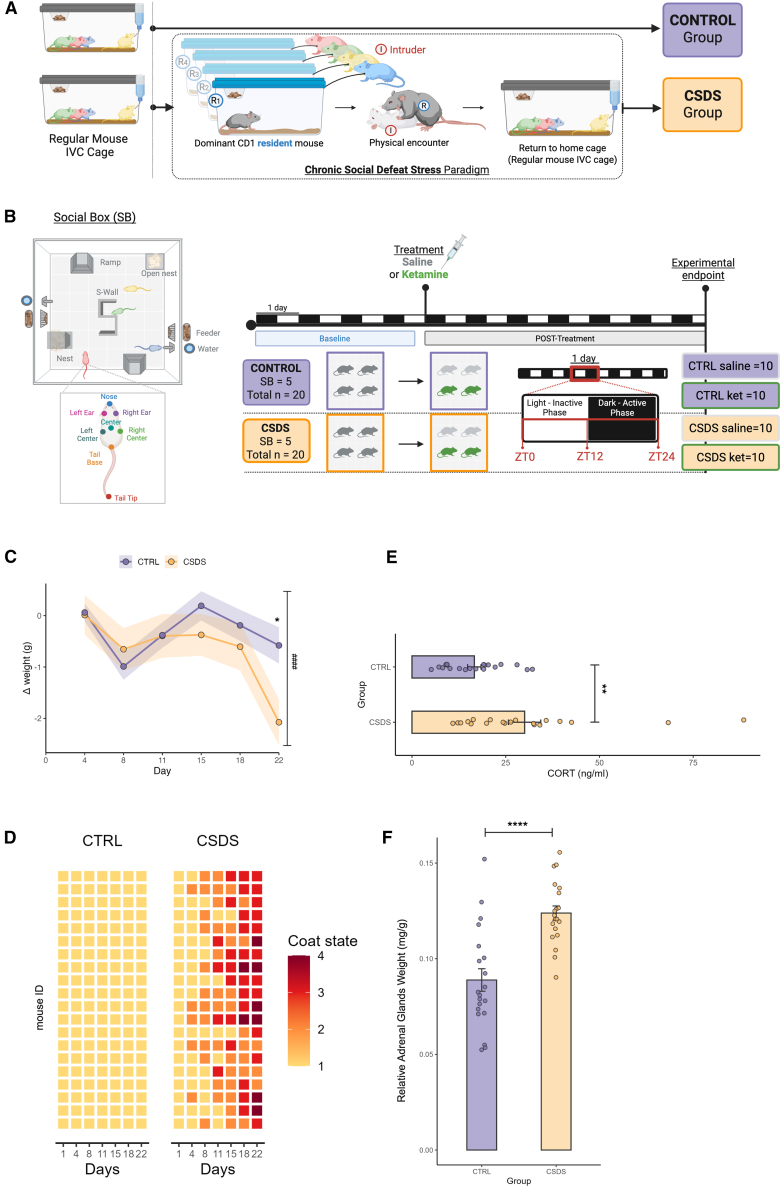
CSDS leads to long-lasting changes in physiological parameters in group-housed male mice (A) Schematic representation of modified CSDS paradigm. Mice, housed in groups of 4, undergo an acute defeat and are then regrouped in their home cage (regular mouse IVC cage). This is repeated for 21 consecutive days. Control mice are group-housed throughout the 21 days in regular mouse IVC cages. (B) Left: The 60 × 60 cm SB arena containing an S wall, two ramps, one closed nest, one open nest, two food feeders, and two water sources. Each SB houses 4 mice. 8 body parts per mouse are tracked with DeepLabCut. Right: experimental timeline of the mice in the SB. Following the completion of the CSDS paradigm (21 days), CTRL (violet) and CSDS (orange) mice were housed in the SB for 4 days (4 active and 4 inactive phases). On the 4^th^ day, they received a single dose of ketamine (green) or saline (light gray) 1 h before the onset of the dark phase. Mice were then housed and recorded for another 7 days in the SB. (C) Changes in body weight across CSDS days. Data represent mean ± SEM, *n* = 20 per condition. Data were analyzed using linear mixed-effect models with group, day, and their interaction as fixed effects, and mouse ID as a random intercept. Post-hoc comparisons were FDR-adjusted. (D) Coat state across the 21 days of CSDS. Rows indicate single mouse IDs, separately for CTRL and CSDS groups. Values close to 4 (orange, red) indicate severe deterioration in coat state. (E) Plasma CORT levels at experimental endpoint in CTRL and CSDS animals. Data represent mean ± SEM. Unpaired *t-test*, two-tailed. (F) Adrenal gland weights normalized to mouse body weights at experimental endpoint. Data represent mean ± SEM. Unpaired *t* test, two-tailed. ∗*p* < 0.05, ∗∗*p* < 0.01, ∗∗∗∗*p* < 0.0001, and ####*p* < 0.0001.

Chronic social defeat is a well-established paradigm that leads to long-lasting physiological, behavioral, and molecular changes in male mice. Here, we used this paradigm to perform an in-depth characterization of the effects of chronic stress, using the SB (Figures 1A and 1B). The SB is an enriched, semi-ethological environment that enables continuous and automated location tracking and behavioral classification of group-housed mice over several days, spanning both active (dark) and inactive (light) phases. The experiment included 10 independent groups, each composed of four male mice (total *n* = 40). Half of the SB groups (5 SB, corresponding to 20 mice) were exposed to CSDS, while the remaining five groups (5 SB, corresponding to 20 mice) served as controls, ensuring balanced representation of stressed and non-stressed conditions. Following CSDS, mice exhibited hallmark stress phenotypes, including significant loss in body weight (group: F(1,38) = 0.67, *p* > 0.05; day: F(5,190) = 15.58, *p* < 0.0001; group x day: F(5,190) = 6.25, *p* < 0.0001), progressive deterioration of their coat state, elevated basal (AM) corticosterone (CORT) levels (group: t(38) = 2.87, *p* = 0.0065), as well as increased weight of the adrenal glands (group: t(38) = 5.03, *p* < 0.0001) (Figures 1C–1F), confirming long-lasting physiological effects of chronic stress.

**Figure 2:**
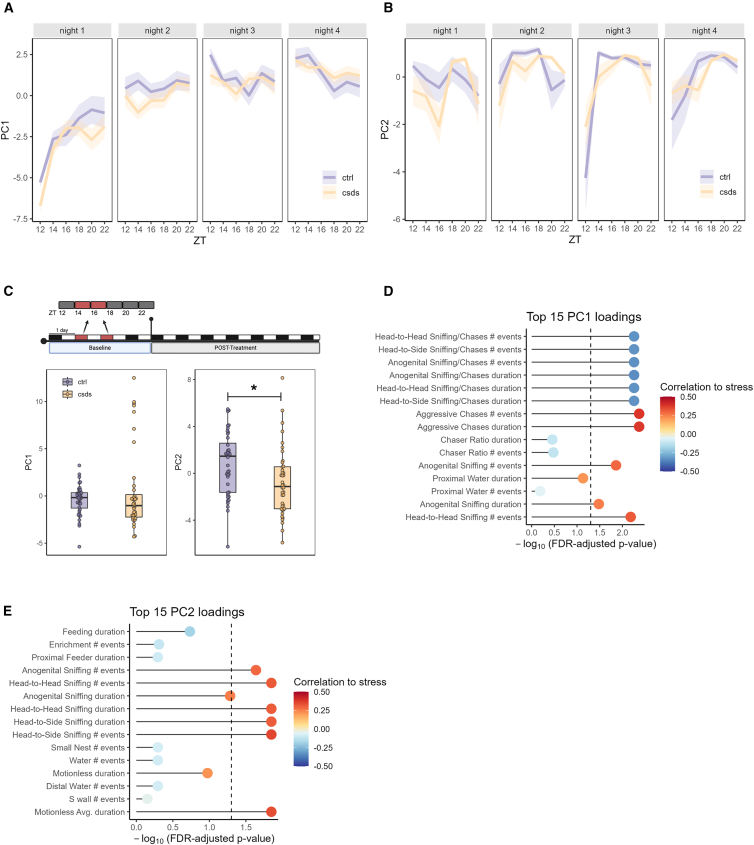
Longitudinal analysis of complex social behaviors in group-housed male mice identifies critical time windows of stress-related phenotypes See also Figures S1–S4. (A) Evolution of PC1(A) and PC2 (B) in CTRL (violet) and CSDS (orange) mice across 2-h intervals during the dark phase, separately for four nights. The x axis represents the zeitgeber time (ZT), where ZT12 indicates the onset of the dark phase. Data represent mean ± SEM; *n* = 20 per condition. Data were analyzed using linear mixed-effect models with group, day, timebin, and their interactions as fixed effects, and mouse ID as a random intercept. PC1. Class: F(1,38) = 0.85, *p* = 0.36; day: F(3,874) = 181, *p* < 0.0001; timebin: F(3,874) = 2.58, *p* = 0.024; class x day: F(3,874) = 2.91, *p* = 0.033; class x timebin: F(3,874) = 1.33, *p* = 0.24; day x timebin: F(15,874) = 10.19, *p* < 0.0001; class x day x timebin: F(15,874) = 1.01; *p* = 0.43. PC2. Class: F(1,38) = 0.12, *p* = 0.72; day: F(3,874) = 2.86, *p* < 0.03; timebin: F(3,874) = 13.9, *p* < 0.0001; class x day: F(3,874) = 0.54, *p* = 0.65; class x timebin: F(3,874) = 2.16, *p* = 0.056; day x timebin: F(15,874) = 4.32, *p* < 0.0001; class x day x timebin: F(15,874) = 1.19; *p* = 0.27. (C) Selected time interval for dissecting the stable effects of stress on behavior. Panels show PC1 and PC2 components across the groups. Data are presented as boxplots showing the median, interquartile range, and whiskers; *n* = 20 per condition. Data were analyzed using linear mixed-effect models with group, day, and their interaction as fixed effects, and mouse ID as a random intercept. ∗*p* < 0.05. (D and E) Correlation (Pearson’s) of top 15 PC1 (D) and PC2 (E) loadings to group (CTRL, CSDS) membership. The dashed line indicates -log (FDR-adj *p* value) = 1.25; values on the right of the line indicate behavioral metrics significantly correlated with stress. Color represents direction and intensity of the correlation (using CTRL as reference).

To capture the temporal dynamics of complex social behaviors, we continuously recorded all groups for four days following the completion of the CSDS protocol, capturing both their active and inactive phases. This generated a comprehensive dataset of automatically computed complex behavioral metrics across the entire recording period. Because rodents exhibit their highest activity levels during the dark phase, we focused our analysis on the 12-h active period across the four post-CSDS days, summarizing behavioral metrics within each interval. To obtain an initial overview of behavioral variation across groups and time, we applied Principal Component Analysis (PCA) over these metrics, focusing on the evolution of the two components (PC1 and PC2) explaining the highest variability (18% and 15%, respectively). As shown in Figure S1A, PC1 accounted for most of the variance associated with time (i.e., across the different nights), with clear distinctions between the first night and the subsequent ones, reaching a plateau by the fourth night. However, fitting a linear mixed-effects model (LMEs) revealed no significant effects of stress on either PC1 or PC2. Because aggregating data over 12-h intervals may obscure or dilute subtle stress-related effects, we next summarized behavior in consecutive 2-h windows, every night, to account for differences driven by zeitgeber time (ZT). This revealed complex interactions between ZT, night, and stress (Figures 2A and 2B).

To disentangle these complex interactions, we initially focused on understanding the effects of stress after introduction into the SB arenas, a completely novel environment. Here, we examined differences in PC1 and PC2 during the first 2 h of night 1, when novelty effects are strongest. This revealed a strong effect of stress (Figure S2B), which is masked when analyzing the first night in its entirety (Figure S2A). This pattern is consistent with findings from Bordes et al.,^27^ who reported that in social interaction tests, differences between CSDS mice and controls are most pronounced during the first few minutes, reflecting the impact of the novel environment. Among the behavioral alterations observed, stressed mice exhibited changes in exploratory patterns, reflected by differential engagement with enrichment objects. Although the total duration of exploration remained unaltered, stressed animals showed increased fragmentation of exploratory behavior, as indicated by a higher number of discrete exploration events directed toward specific objects or areas (Figures S2C and S2D). To identify more persistent stress effects beyond novelty-driven changes, we next examined behavior after habituation to the SB. Since 2-h intervals result in low signal-to-noise ratios due to the sparse nature of some behaviors, we performed PCA analysis across larger time intervals (4 h and 6 h) throughout the 4 nights (Figures S1B, S1C, and S3A–S3E), which revealed interesting and distinct behavioral signatures. Closer to the inactive phase (ZT18-24 or ZT20-24), group differences were evident only on the first night, whereas during the first half of the night, group differences were more stable across different nights. This was particularly evident when considering the time interval between ZT14 and ZT18 (Figure S3B). To minimize novelty confounds, we excluded night 1 from subsequent analyses. Previous studies have also consistently reported that mice habituate to the SB within a few days in the absence of external stressors or stimuli, leading to a marked reduction in behavioral variability.^26^ Therefore, our subsequent analyses focused on nights 2 and 3, specifically within the ZT14–18 interval, to further characterize the persistent behavioral alterations induced by CSDS. This shows night-independent effects of stress on cumulative behavior (PC1 group: F(1,38) = 0.88, *p* > 0.05; PC2 group: F(1,38) = 4.36, *p* = 0.043) (Figure 2C). Interestingly, the acute effects observed on exploration during the first night do not persist at these subsequent timepoints. Instead, correlations of the top PC1 and PC2 loadings with stress condition indicate that chronic stress is associated with increased immobility time and more agonistic interactions, such as chasing behaviors (Figures 2D and 2E).

While the active (dark) phase is the period with increased activity in rodents, most behavioral tests performed in other laboratories to assess stress and treatment response are carried out during the inactive (light) phase. Thus, to determine whether stress-related alterations observed throughout the night persist during the day, we analyzed behavior across the light phase (Figures S4A and S4B). Similar to the active phase, ZT strongly influenced behavioral patterns. In this context, the most pronounced differences emerged during the second half of the light phase, particularly between ZT6 and ZT10 (Figure S4C). Stressed mice consistently spent less time in the nest across all 4 days, indicating that rest fragmentation may be a persistent consequence of chronic stress in male mice (Figure S4D).

### Social dominance exacerbates the effects of chronic stress

**Figure 3:**
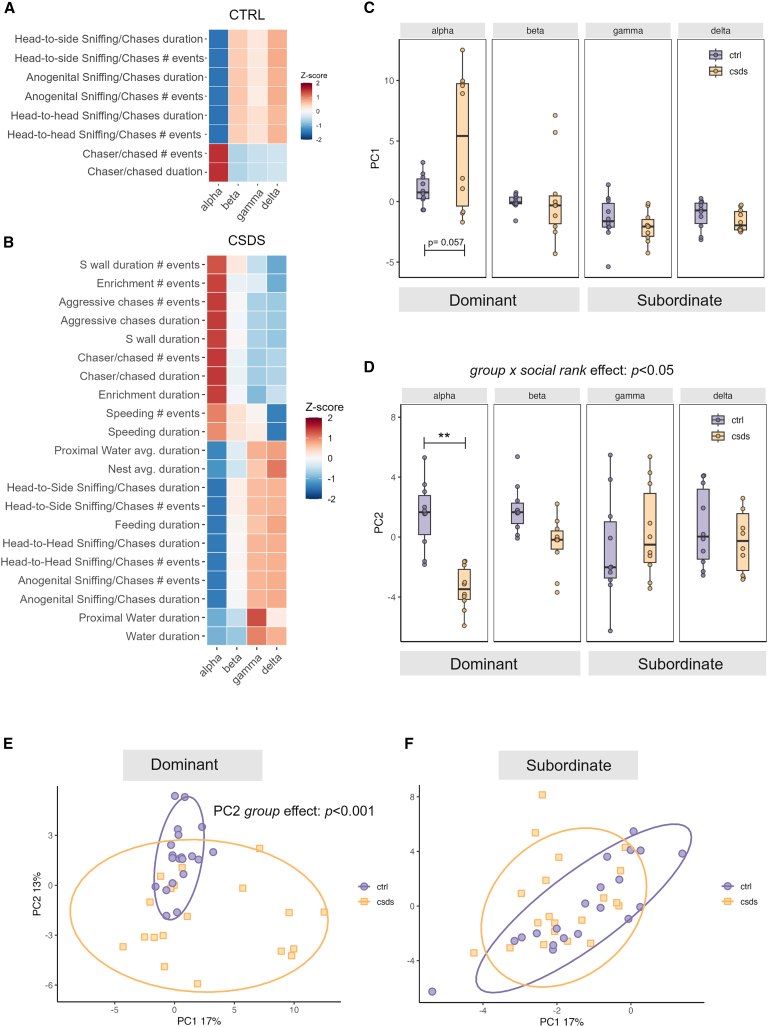
Social dominance exacerbates the effects of chronic stress (A and B) Hierarchical clustering of Z-scored behavioral metrics that significantly correlate with hierarchy rank (alpha = more dominant, delta = more subordinate), separately for CTRL (A) and CSDS (B) mice. (C and D) Boxplot of PC1 (C) and PC2 (D) in CTRL and CSDS mice, by hierarchy rank. Data are presented as boxplots showing the median, interquartile range, and whiskers. Data were analyzed using linear mixed-effect models with group, hierarchy rank, and their interaction as fixed effects, and mouse ID as a random intercept. Post-hoc comparisons were FDR-adjusted. (E and F) PCA plot of dominant (alpha, beta) (E) and subordinate (gamma, delta) (F) mice. Data were analyzed using linear mixed-effect models with group, hierarchy rank, and their interaction as fixed effects, and mouse ID as a random intercept. ∗∗*p* < 0.01.

Social dominance is a critical factor shaping behavioral outcomes and can modulate vulnerability to chronic stress.^10^^,^^11^^,^^12^^,^^13^^,^^14^ Groups of mice exhibit stable social hierarchies that govern access to resources and regulate social interaction dynamics.^28^ Thus, position in the social hierarchy strongly influences some behavioral components and has been linked to distinct susceptibility to chronic stress.^10^^,^^11^^,^^12^ To explore this relationship, we first quantified dominance ranks and then examined how these ranks interact with stress exposure to shape behavior. To do this, we calculated the cumulative normalized David’s score (normDS) during nights 2–4, a widely used metric for quantifying dominance relationships in group-housed rodents. Based on normDS, each mouse was assigned a social rank, ranging from alpha (most dominant), beta, gamma, to delta (most subordinate). This ranking provided a framework to assess whether stress-related behavioral changes vary systematically across hierarchy positions. We looked at behavioral readouts significantly correlated with position in the hierarchy, separately for CTRL and CSDS mice. In both groups, alpha males exhibit a clearly distinct behavioral profile from the other mice. However, the nature of these differences diverged between conditions. While in the CTRL group the behaviors associated with increased dominance are exclusively related to agonistic interactions, in the CSDS group dominance is also associated with more fragmented interactions with the environment, decreased resting (average time spent in the closed nest), and decreased feeding and drinking behavior (Figures 3A and 3B). Furthermore, in the CSDS group only, beta males exhibit an intermediate profile between the alpha and the more subordinate animals. This finding suggests that chronic stress amplifies behavioral stratification within the group’s hierarchy.

Next, we applied PCA again across all behavioral readouts. This analysis aimed to determine whether the overall behavioral profile (summarized by PC1 and PC2) was significantly different between stressed and control animals across hierarchy ranks. As shown in Figures 3C and 3D, PC2 recapitulates an important interaction effect between social hierarchy and stress (group: F(1,32) = 5.36, *p* = 0.027; hierarchy rank: F(3,32) = 1.2, *p* > 0.05; group x hierarchy rank: F(3,32) = 3.67, *p* = 0.0221). Because of their similar behavioral profiles, and to increase statistical power, alpha and beta mice were pooled together and considered “*dominant*” individuals, while gamma and delta were grouped together as “*subordinates.*” This grouping revealed a clear pattern, where differences emerge between control and stressed animals occupying high rank positions (dominant) (PC1 group: F(1,18) = 1.81, *p* > 0.05; PC2 group F(1,18) = 22.84, *p* = 0.0001), while no significant difference between control and stressed mice was observed in the subordinate group (PC1 group: F(1,18) = 1.48, *p* > 0.05; PC2 group F(1,18) = 0.13, *p* > 0.05) (Figures 3E and 3F). As stress-related behavioral effects were detected only in dominant mice, subsequent analyses focused on this subgroup to assess treatment effects.

**Figure 4:**
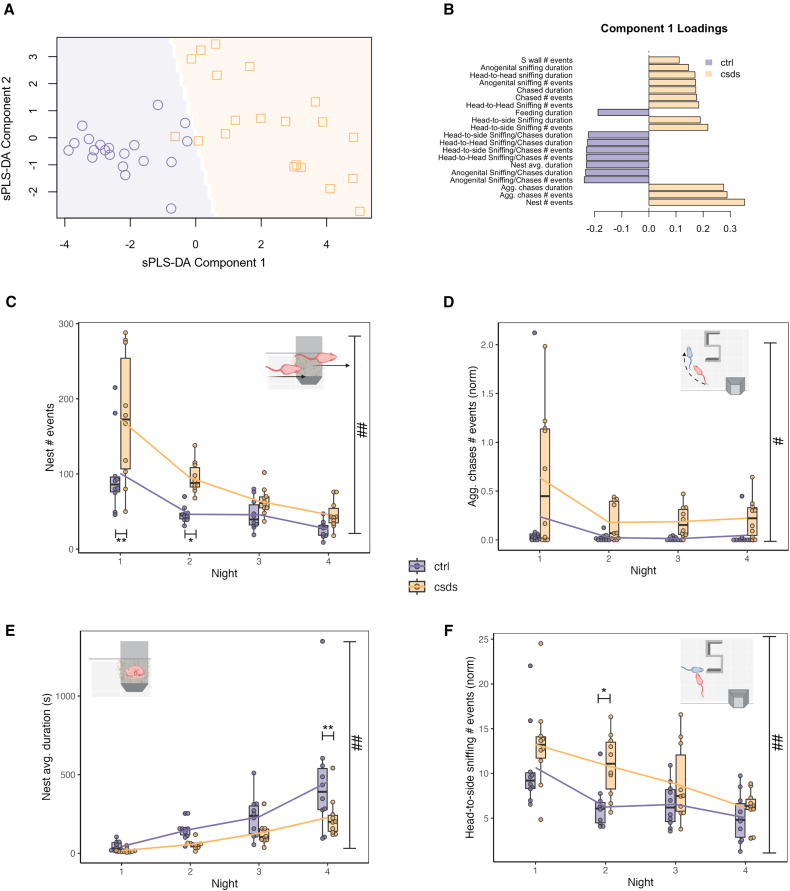
Dominant mice show alterations in specific behaviors following chronic stress (A) sPLS-DA plot shows the separation between groups based on components 1 and 2, in dominant mice. CTRL (violet), CSDS (orange). (B) Component 1 loadings. The size of the bar represents the contribution of that behavioral metric to component 1; the direction represents positive (right) or negative (left) contribution. (C–F) Evolution of number of nest events (C) (class: F(1,18) = 14.60, *p* = 0.0013; day: F(3,54) = 29.24, *p* < 0.0001; class x day: F(3,54) = 2.55, *p* = 0.064), number of aggressive chases (D) (class: F(1,18) = 5.11, *p* = 0.036; day: F(3,54) = 4.88, *p* = 0.0045; class x day: F(3,54) = 0.61, *p* > 0.05), average nest event duration (E) (class: F(1,18) = 8.84, *p* = 0.0081; day: F(3,54) = 18.18, *p* < 0.0001; class x day: F(3,54) = 1.77, *p* > 0.05), and number of head to side sniffing events (F) (class: F(1,18) = 8.66, *p* = 0.0087; day: F(3,54) = 11.97, *p* < 0.0001; class x day: F(3,54) = 0.94, *p* > 0.05) across 4 nights, in dominant CTRL (violet) and CSDS (orange) animals. Data are presented as boxplots showing the median, interquartile range, and whiskers; *n* = 10 per condition. Data were analyzed using linear mixed-effect models with group, day, and their interaction as fixed effects, and mouse ID as a random intercept. Post-hoc comparisons were FDR-adjusted. ∗*p* < 0.05, ∗∗*p* < 0.01, #*p* < 0.05, and ##*p* < 0.01.

To further dissect which behavioral features drive this separation, we trained a sparse Partial Least Squares Discriminant Analysis (sPLS-DA) classifier using the behavioral readouts of the dominant mice only. Figure 4A shows that a nearly perfect separation is achieved between the classes, with component 1 driving the separation. When examining the component 1 loadings, we confirm the behavioral profile that was captured by PCA as well. Indeed, stress seems associated with rest fragmentation, increased chasing and sniffing behavior, as well as decreased time in the feeding area (Figure 4B). Figures 4C–4F report some examples of behaviors that differ between the control and stressed groups, across the 4 nights. Although behavior evolves over time, these differences remain remarkably consistent, reinforcing the notion that dominance is associated with increased stress vulnerability.

### A single ketamine administration mitigates the behavioral effects of chronic stress

**Figure 5:**
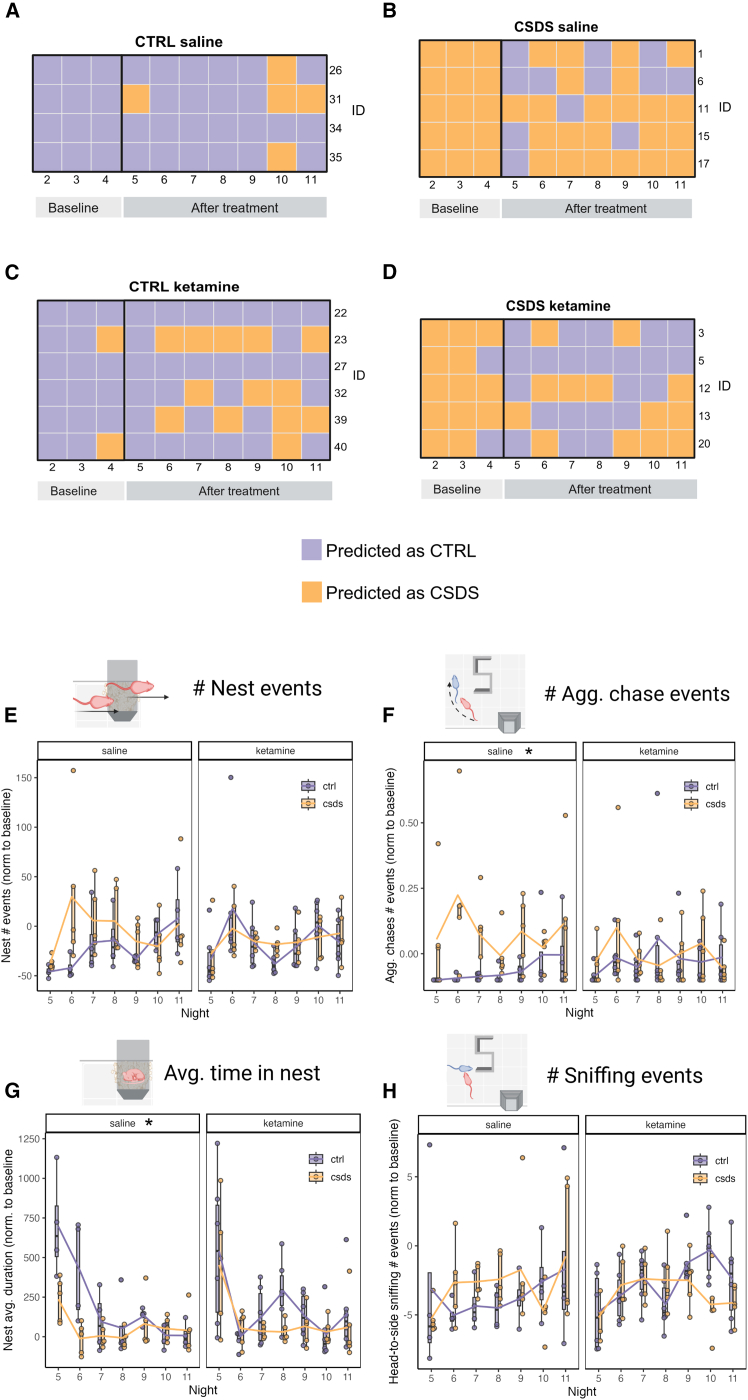
A single ketamine administration mitigates the effects of chronic stress at the behavioral level See also Figure S5. (A–D) Predictions based on the previously trained sPLS-DA, throughout 10 days in the SB. For each day, dominant mice were predicted as either CTRL (violet) or CSDS (orange), for each condition: CTRL saline (A), CSDS saline (B), CTRL ketamine (C), and CSDS ketamine (D). Data were analyzed using generalized linear mixed-effect models with group, treatment, and their interaction as fixed effects, and mouse ID as a random intercept. Post-hoc comparisons were FDR-adjusted. (E and F) Evolution of number of nest events (E), number of aggressive chases (F), average nest event duration (G), and number of head-to-side sniffing events (H) across the 7 nights following treatment administration (normalized to baseline values). Data are presented as boxplots showing the median, interquartile range, and whiskers; *n* = 5 per condition. Data were analyzed using linear mixed-effect models with group, treatment, and their interaction as fixed effects, and mouse ID as a random intercept. Post-hoc comparisons were FDR-adjusted. ∗*p* < 0.05.

A single ketamine administration has been shown to produce rapid and long-lasting antidepressant effects in patients afflicted by depression as well as in chronically stressed mice. Building on this evidence, we examined whether ketamine could rescue stress-induced behavioral alterations in our SB paradigm. To do so, we leveraged the previously trained sPLS-DA classifier to track behavioral changes over the 7 days following ketamine administration. First, we confirmed that the distribution of the 3 sPLS-DA components in the saline group did not change significantly from the 4 baseline days (Figure S5A). Next, we looked at baseline night 4, which was not included in the training dataset. As expected, this maintained a prediction in line with the other baseline nights, suggesting a good predictive validity of the model (Figures 5A–5D). Having established model reliability, we then applied it to post-treatment data. To do so, we used the classifier to obtain a prediction for each mouse, for each of the 7 post-treatment days. Namely, each mouse was predicted as either CTRL or CSDS, based on the score of the 3 sPLS-DA components. This approach allowed us to quantify whether ketamine shifts stressed mice toward a control-like behavioral profile. As expected, predictions in the saline group were consistent with the baseline group assignments, with significant differences between CTRL and CSDS throughout the 7 days (group: c^2^(1) = 4.73, *p* = 0.029; treatment: c^2^(1) = 0.5, *p* > 0.05; group x treatment: c^2^(1) = 3.54, *p* = 0.059. FDR-adjusted post-hoc comparison, saline group, ctrl-csds: z = 2.84, *p* = 0.0089). Conversely, no statistically significant difference was observed between dominant CTRL and CSDS mice that received ketamine, suggesting a long-lasting rescuing effect of ketamine at the behavioral level (group: c^2^(1) = 4.73, *p* = 0.029; treatment: c^2^(1) = 0.5, *p* > 0.05; group x treatment: c^2^(1) = 3.54, *p* = 0.059. FDR-adjusted post-hoc comparison, ketamine group, ctrl-csds: z = 0.47, *p* > 0.05) (Figures 5C and 5D). Direct comparison further confirmed that a single ketamine administration rescued the behavioral profile of stressed mice to levels comparable to saline-treated controls, consistent with a normalization of the stress-induced phenotype (group: c^2^(1) = 4.73, *p* = 0.029; treatment: c^2^(1) = 0.5, *p* > 0.05; group x treatment: c^2^(1) = 3.54, *p* = 0.059. FDR-adjusted post-hoc comparison, csds ketamine—ctrl saline: z = 1.11, *p* > 0.05) (Figure S5B). Importantly, this effect was not confounded by changes in social hierarchy position, as normDS remained stable across treatment conditions (Figure S5C). Consistent with the prediction results, examination of specific behaviors altered in the stressed group recapitulated these findings. In the saline group, a statistically significant difference is observed between CTRL and CSDS mice concerning the number of aggressive chases and the average time spent in the closed nest (Figures 5E–5H). These differences are not observed in mice that received ketamine, suggesting that this compound may mitigate the deleterious effects of chronic stress on rest fragmentation, as well as the deficits in agonistic behaviors. Taken together, these results indicate that ketamine restores both global complex behavioral signatures and specific stress-sensitive behaviors in dominant mice.

It should also be noted that almost all mice were classified as CTRL immediately after compound administration (both saline and ketamine). We attribute this transient shift to a marked increase in time spent inside the nest following injection, likely reflecting an acute stress response to the procedure itself. Nest hiding is consistent with the behavior typically observed in mice after exposure to sudden stressors. This brief but pronounced change drives all sPLS-DA components toward CTRL-like values. Although short-lived, this effect highlights the sensitivity of our classifier to acute perturbations and underscores the importance of considering procedural stress in behavioral analyses.

### Ketamine administration leads to long-lasting, region-specific effects of gene expression in dominant male mice

**Figure 6:**
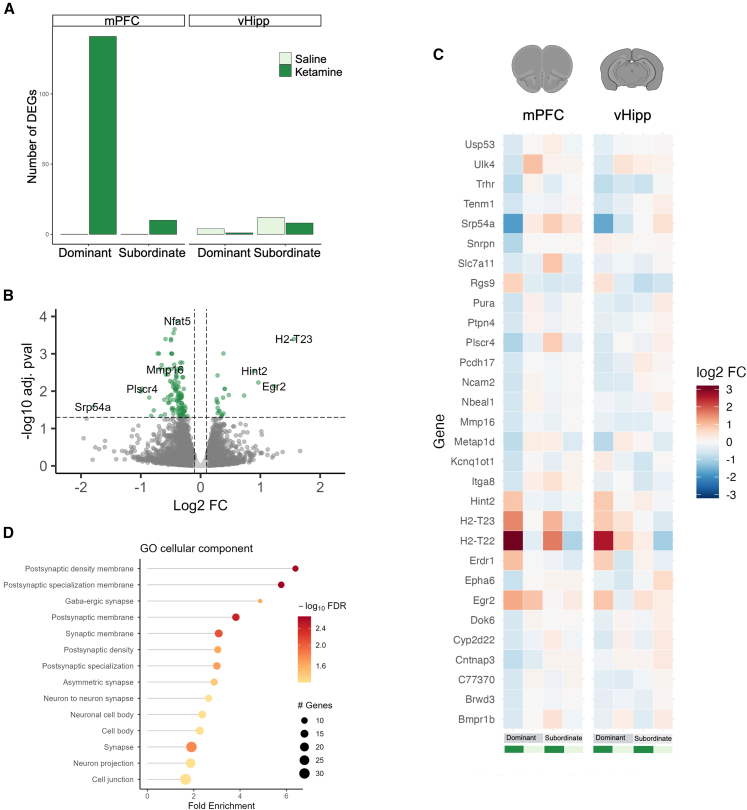
Ketamine administration leads to long-lasting, region-specific effects of gene expression in dominant male mice See also Figure S6. (A) Bar plot represents number of DEGs per condition: hierarchy rank (dominant, subordinate), treatment (saline, ketamine) within each region (mPFC, vHipp). (B) Volcano plot represents LFC of mPFC genes in ketamine-treated, dominant mice. Orange dots represent genes with absolute LFC >0.1 and FDR-adjusted *p* value <0.05. (C) Heatmap of top DEGs (ranked by absolute LFC), separately for mPFC and vHipp. Each column indicates a specific rank (dominant, subordinate) and treatment (saline, ketamine) combination. Color represents the intensity and direction of the LFC. (D) GO enrichment for category “cellular component,” using the 141 DEGs identified in the mPFC of ketamine-treated, dominant mice. Position along the x axis indicates the fold enrichment for that term; color indicates the -log of FDR-adjusted *p* value.

The mPFC and vHipp represent central nodes in stress-related circuitry, supporting functions such as emotional regulation, cognitive flexibility, social behavior, and treatment response. While many studies have focused on the rapid antidepressant effects of ketamine in these brain regions, data on sustained molecular changes remain scarce, and the role of social hierarchy in shaping these effects is largely unexplored. To address this gap, we performed bulk RNA sequencing on all mice that underwent the behavioral assessment (*n* = 40), followed by differential expression analysis in the mPFC and vHipp, examining the influence of stress across social hierarchy and treatment groups. Our analysis revealed a striking pattern: 141 unique DEGs were identified in dominant mice treated with ketamine within the mPFC, but not in the vHipp (Figure 6A). Most of these genes were downregulated in stressed animals, while the few upregulated ones displayed substantial log fold changes (LFCs) (Figure 6B). The gene with the highest absolute LFC, H2-T22, was validated in both brain regions by qPCR, showing strong and significant correlation between the RNA-seq and qPCR values (Figures S6A and S6B).

Upon closer inspection, we observed that the top 30 DEGs ranked by absolute LFC exhibited similar directional changes in both brain regions, though effects were markedly stronger in the mPFC (Figure 6C). Notably, these transcriptional changes were specific to dominant mice treated with ketamine, suggesting that social hierarchy critically shapes the transcriptional landscape in these brain regions and modulates molecular responses to treatment. Finally, to gain functional insight, we performed gene enrichment analysis on the 141 DEGs of the dominant, ketamine-treated mice in the mPFC (Figure 6D). This revealed enrichment in specific cellular compartments central to synaptic signaling, consistent with known mechanistic targets of ketamine. These findings indicate that synaptic remodeling might be a potential substrate for ketamine’s sustained effects. Overall, even after 7 days after administration, complex interaction effects between stress and ketamine persist at the gene expression level, with pronounced changes in the mPFC and more modest effects in the vHipp. Future studies should dissect the functional role of these genes and clarify how hierarchy-dependent molecular signatures translate into behavioral resilience.

## Discussion

Chronic stress has long been recognized as a major risk factor for the development of psychiatric disorders, including depression, anxiety, and PTSD.^1^^,^^2^^,^^3^ Despite extensive preclinical research using rodent models, the mechanisms underlying individual variability in maladaptive stress responses remain poorly understood. One contributing factor to this gap may be the reductionist nature of traditional behavioral testing, which typically relies on brief, isolated assessments conducted in artificial settings. Such paradigms neglect critical dimensions of behavior such as social context, daily rhythms, and longitudinal adaptation. In this study, we implemented the SB, an automated and high-throughput system for continuous behavioral phenotyping in group-housed mice, to investigate the effects of chronic stress and a single ketamine administration over an 11-day period, spanning both active (dark) and inactive (light) phases. This approach allows for minimal experimenter intervention, longitudinal monitoring across both the light and dark phases, and inference of complex social behaviors, thus providing a more comprehensive view of stress-related behavioral adaptations. It also enables the identification of subtler and temporally specific changes that may be obscured in short-duration tests. Our results reveal that the behavioral impact of chronic stress varies significantly throughout the 24h light-dark cycle. During the active phase, group differences were most pronounced during the early night (ZT 14–18), when stressed mice displayed increased fragmentation of resting behavior and elevated agonistic interactions. These findings suggest that stress disrupts multiple behavioral domains during the period of peak activity, when animals are typically engaged in exploration and social interaction. During the inactive phase, we similarly observed stress-induced rest fragmentation, particularly between ZT 6–10. Notably, this period coincides with the time window during which most standard behavioral tests are typically conducted. This raises important methodological considerations, as testing during the light phase may capture only some deficits in behavior, and different timing choices could contribute to the variability and inconsistencies often reported across laboratories. Furthermore, these time windows correspond to twilight transitions, when light conditions shift between day and night (ZT 14–18 and ZT 6–10). Because mice are crepuscular animals, these periods represent ecologically relevant, high-risk windows in which they are most vulnerable to predation and exhibit heightened anxiety. This underscores the significance of our findings, as stress-related behavioral disruptions during these critical transitions may reflect an amplified threat-processing state, revealing a biologically meaningful link between circadian timing and stress susceptibility. Most importantly, our data point to the importance of longitudinal, semi-ethologically relevant behavioral testing. While traditional tests only provide a “*snapshot*” of behavior, which is likely influenced by novelty, social isolation, and experimenter handling, our SB system enables us to capture the natural dynamics and fluctuations of behavior, including daily rhythms and adaptation.

After establishing the influence of daily rhythms, we next examined another critical factor shaping stress vulnerability: social hierarchy, which introduces a social dimension to these behavioral patterns. Social structure plays a critical role in shaping stress responses and coping mechanisms. However, most behavioral tests are restricted to examining dyadic interaction, and therefore fail to capture more complex social dynamics. In both rodents and primates, including humans, social hierarchy and dominance rank are key in shaping an individual’s behavior in relation to the environment and its conspecifics, as well as its stress response. Despite this, the interaction between social hierarchy and chronic stress remains relatively underexplored. To address this gap, we quantified social hierarchies in group-housed mice using David’s score,^29^ enabling us to test whether high dominance rank amplifies or mitigates stress-induced behavioral changes. Our data indicate that dominant mice exhibit greater vulnerability to the effects of chronic stress, showing marked deficits in social interactions and rest fragmentation throughout the 11 days in the SB. These findings are consistent with the work from Larrieu et al.,^11^ who also reported that CSDS is associated with increased stress susceptibility in dominant mice, reflected by elevated anxiety behaviors, while no differences were observed in the subordinate group. This pattern reinforces the notion that dominance confers heightened sensitivity to social defeat stress, either by directly shaping stress response, or because dominance may reflect pre-existing traits that influence stress response. While our design cannot disentangle between these two possibilities, it nevertheless underscores the importance of evaluating the interplay between stress and hierarchy when using mouse models of neuropsychiatric disorders. One potential explanation for this increased vulnerability could originate from the nature of the stress paradigm. More specifically, dominant mice may be particularly challenged by the repeated defeat session, while subordinate animals, more used to defeat in the social setting, exhibit adaptive coping strategies that confer resilience to these stress conditions. This interpretation aligns with findings by Fan et al.,^30^ showing that forced social status loss is associated with depressive-such as behaviors, as indicated by increased immobility in the forced swim test (FST), which can be reversed by ketamine administration. Additionally, an association between increased stress levels and high social rank has also been reported in other species where despotic dominance is maintained through frequent physical reassertion of dominance, or when hierarchy is unstable.^13^^,^^31^ Finally, in humans, social status threat is a recognized risk factor for psychiatric conditions like major depression and substance abuse disorders, particularly in men.^32^^,^^33^ It’s worth noting that other studies have concluded that subordinate, rather than dominant mice, exhibit increased vulnerability to stress.^10^ These discrepancies may arise from differences in the stressors employed,^12^ as well as from the methodological approaches used to quantify social hierarchy and stress vulnerability. Whereas many prior studies rely on experimenter-driven dominance assays such as the tube test and single-endpoint behavioral readouts, our SB approach quantifies hierarchy and social interactions continuously within stable groups living in an enriched, naturalistic environment. While most studies use social interaction as a single metric to evaluate vulnerability to stress, our naturalistic and longitudinal approach may provide a more comprehensive view, possibly capturing different aspects of the stress-hierarchy interaction. Finally, we want to clarify that our analysis did not aim to generate the classical binary split into susceptible or resilient animals. Rather, we suggest that specific subgroups (namely dominant mice) are more vulnerable to the effects of chronic social defeat, as evidenced by stronger behavioral disruptions. Consistently, our analytical strategy focused on directly comparing stressed vs. control animals within the dominant and subordinate groups.

Overall, this evidence underscores the translational relevance of our findings and highlights the need to integrate social hierarchy as a core variable in stress research. While our findings offer valuable insights, future studies should determine whether hierarchy-stress interactions extend to other chronic stress paradigms and experimentally manipulate group hierarchy to directly test its causal role in stress vulnerability. Additionally, a limitation of the present study is that hierarchy was calculated using behavioral measures that also overlap (through only partially) with some of the outcome variables, and baseline measurements were not available to fully disentangle pre-existing rank-related differences from stress-induced changes. Future studies incorporating baseline assessments would help separate these effects more clearly and further strengthen the interpretation of hierarchy-dependent stress responses. Finally, our experiments were conducted in male mice, which limits generalizability across sexes. Notably, the CSDS paradigm is inherently male-biased, as female mice rarely exhibit the sustained aggression required for repeated defeat. To address this gap, future work should incorporate modified versions of CSDS for females or alternative paradigms, such as chronic unpredictable mild stress, to investigate hierarchy-dependent stress vulnerability and treatment response in a sex-specific manner.

With hierarchy identified as a factor associated with stress vulnerability, we next asked whether pharmacological interventions could mitigate these effects. Ketamine, an NMDA receptor antagonist, offers a unique opportunity to address this question given its rapid and sustained antidepressant properties. Over the past decade, ketamine has transformed psychiatric treatment, yet the mechanisms underlying this dual timing of effect have not been fully elucidated. Moreover, substantial interindividual variability in treatment response persists, with some individuals experiencing robust and durable benefits, while others show limited effects. To explore this, we examined whether ketamine could reverse stress-induced behavioral alterations in our SB paradigm, providing a high-resolution view of its effects over time (up to 7 days after administration). Our data reveal that dominant mice previously exposed to chronic stress exhibit behavioral recovery following ketamine administration, displaying activity and social patterns comparable to control animals. In contrast, stressed dominant mice receiving saline maintained behavioral signatures of chronic stress, characterized by persistent rest fragmentation and altered agonistic interactions, throughout the remaining days in the SB. These findings suggest that ketamine attenuates the long-term behavioral consequences of chronic social stress, restoring both rest structure and social behavior toward baseline levels. It should be noted that ketamine also partially shifts the behavioral pattern in control animals, which increases the chance of misclassification. However, specific behavioral features remain uniquely and consistently rescued in stressed mice, supporting the conclusion that ketamine selectively mitigates stress-induced deficits. Although our sample size precluded detailed day-by-day mapping of ketamine’s behavioral trajectory, our results indicate that its therapeutic effects can be captured in the SB for up to seven days after treatment administration. This is in contrast with our previous findings using the FST, where ketamine-induced reductions in immobility were only detected for 5 days post-injection and disappeared by day 7.^26^ Our new findings highlight the greater sensitivity of the SBs, which captures sustained and ecologically relevant behavioral improvements beyond the temporal window detected by conventional tests. Nevertheless, future studies should include larger cohorts to enable fine-grained temporal analysis of ketamine’s effects at the behavioral level, and dissection of the implications of single dominance ranks in treatment response.

This sustained behavioral recovery prompted a critical question: do these improvements reflect underlying molecular adaptations, and does social hierarchy modulate this response? To address this, we performed bulk mRNA sequencing of the mPFC and vHipp, identifying DEGs between control and stressed mice across social hierarchy ranks and treatment conditions. In subordinate animals, as well as in saline-treated mice, the number of DEGs was very limited in both regions. In contrast, we identified 141 unique DEGs in the mPFC of dominant mice treated with ketamine, a subset that showed similar but attenuated fold-change patterns in the vHipp. These hierarchy-dependent transcriptional changes suggest that ketamine’s molecular effects are shaped by social context, reinforcing the behavioral evidence for rank-specific vulnerability and recovery. Gene enrichment analysis revealed that these DEGs were significantly enriched in synaptic and postsynaptic compartments. This is consistent with well-known targets of ketamine.^16^ By linking behavioral recovery to synaptic pathways, our results highlight a convergence between molecular-level plasticity and social context, offering a mechanistic basis for hierarchy-dependent treatment outcomes. Together, these findings indicate that a single dose of ketamine induces persistent transcriptional remodeling in stress-sensitive brain circuits that linger seven days post-administration, and that these effects are modulated by social hierarchy rank, providing molecular evidence supporting the sustained behavioral improvements observed in dominant mice.

Future studies should build on these findings by integrating the identified region-level transcriptional signatures with cell-type-resolved and spatially informed approaches, enabling the dissection of the specific cellular populations and circuit-level interactions through which these molecular programs contribute to stress resilience and antidepressant effects.

Overall, the key advance of this study is that it integrates continuous, naturalistic group-housed behavioral phenotyping across circadian phases with region-resolved transcriptomics to show that both stress vulnerability and ketamine response differ as a function of social hierarchy position, with distinct molecular signatures in the mPFC versus hippocampus.

### Limitations of the study

Our study suggests that social dominance confers heightened sensitivity to social defeat stress. However, due to the nature of the CSDS paradigm, female mice were not included in the study. Future work should evaluate whether the current findings generalize to both sexes. Additionally, our study design cannot fully disentangle whether the association between increased vulnerability to social stress and dominance is a direct result of dominance on the stress response, or whether dominant mice possess pre-existing traits that influence the stress response. Finally, our current experimental design and number of animals allowed us to only separate the mice into dominant and subordinate categories when evaluating the effects of ketamine. Further research is needed to fully separate the effects of the single hierarchy ranks and to increase the temporal resolution when evaluating treatment efficacy.

## Resource availability

### Lead contact

Requests for further information and resources should be directed to and will be fulfilled by the lead contact, Juan Pablo Lopez (jpablo.lopez@ki.se).

### Materials availability

This study did not generate new unique reagents.

### Data and code availability

#### Data

• Bulk RNA-seq data have been deposited at Gene Expression Omnibus (GEO) as “GEO: GSE325386” and are publicly available as of the date of publication.
• All data reported in this paper will be shared by the lead contact upon request.

#### Code

• All original code has been deposited at GitHub and is publicly available at https://github.com/LopezLab-KI/Behavioral_analysis_SB as of the date of publication.

#### Additional information

Any additional information required to reanalyze the data reported in this paper is available from the lead contact upon request.

## Acknowledgments

We thank Lucas Dumargne, Cornelia Flachskamm, and Rainer Stoffel, as well as the animal facilities and staff at the Max Planck Institute of Psychiatry and the Animal Behavior Core Facility (ABCF) at 10.13039/501100004047Karolinska Institutet for their technical assistance and support. The computations/data handling were enabled by resources provided by the National Academic Infrastructure for Supercomputing in Sweden (NAISS), partially funded by the Swedish Research Council through grant agreement no. 2022-06725. The authors acknowledge support from the National Genomics Infrastructure in Genomics Production Stockholm funded by 10.13039/501100009252Science for Life Laboratory, the 10.13039/501100004063Knut and Alice Wallenberg Foundation and the Swedish Research Council, and NAISS/10.13039/501100015701Uppsala Multidisciplinary Center for Advanced Computational Science for assistance with massively parallel sequencing and access to the 10.13039/501100015701UPPMAX computational infrastructure. JPL receives research funding from the 10.13039/501100003748Swedish Society for Medical Research (SSMF) (grant no. SG-22-0204), the Swedish Research Council (VR) (grant no. 2023-02499), the 10.13039/501100003792Swedish Brain Foundation (Hjärnfonden) (grant nos. FO2023-0313 and FO2024-0038), the Strategic Research Area Neuroscience (StratNeuro), and the 10.13039/501100000781European Research Council (ERC) (grant no. 101116064) through a Starting Grant.

## Author contributions

Conceptualization: S.G. and J.P.L.; methodology: S.G. and J.P.L.; data analysis: S.G. and J.P.L.; software: S.G., X.J., C.S.C., T.B., D.U., and Y.S.; investigation: S.G., A.K., and E.C.P.; validation: S.G., O.B., M.M.H., F.S.Z., and E.C.P.; resources: A.C. and J.P.L.; data curation: S.G., X.J., C.S.C., O.B., M.M.H., and F.S.Z.; visualization: S.G. and P.F.; supervision: J.P.L.; writing – original draft: S.G. and J.P.L.; writing – editing: All authors; project administration: J.P.L. funding acquisition: J.P.L.

## Declaration of interests

All authors have confirmed that they have no financial or non-financial conflicts that could affect their objectivity or the content of the article.

## Declaration of generative AI and AI-assisted technologies in the writing process

During the preparation of this work, the authors used ChatGPT in order to assist with writing and debugging the scripts used for data analysis. After using this tool or service, the authors reviewed and edited the content as needed and take full responsibility for the content of the publication.

## STAR★Methods

### Key resources table

**Table:** 

REAGENT or RESOURCE	SOURCE	IDENTIFIER
**Antibodies**		

CORT double antibody 125I RIA kit	MP Biomedicals Inc.	Cat# 07120102; RRID: AB_2783720

**Chemicals, peptides, and recombinant proteins**		

(R,S)-ketamine Hydrochloride - Ketaset	Zoetis	N/A

**Critical commercial assays**		

miRNeasy Micro kit	Qiagen	Cat # 217084
High-Capacity cDNA reverse transcription kit	Thermo Fisher Scientific	Cat # 4368814
QuantiFast SYBR green PCR kit	Qiagen	Cat # 204056

**Deposited data**		

Bulk RNA sequencing data	This Manuscript	GEO: GSE325386
Code for behavior classifiers	This Manuscript	https://github.com/LopezLab-KI/Behavioral_analysis_SB

**Experimental models: Organisms/strains**		

Mouse: CD-1 (ICR) male mice	MPI Biochemistry (Germany)	N/A

**Oligonucleotides**		

*Gapdh* fwd primer qpcr	Integrated DNA Technologies	CCATCACCATCTTCCAGG AGCGAG
*Gapdh* rev primer qpcr	Integrated DNA Technologies	GATGGCATGGACTGTGG TCATGAG
*Rpl13* fwd primer qpcr	Integrated DNA Technologies	CACTCTGGAGGAGAAAC GGAAGG
*Rpl13* rev primer qpcr	Integrated DNA Technologies	GCAGGCATGAGGCAAAC AGTC
*H2-T22* fwd primer qpcr	Integrated DNA Technologies	GGAGCAGGAGGAAGCAG ATA
*H2-T22* rev primer qpcr	Integrated DNA Technologies	TTGGGTTTTCGTTCAGGG TG

**Software and algorithms**		

QuantStudio Real-time PCR software	Applied Biosystems	https://www.thermofisher.com/se/en/home/tech nical-resources/software-downloads/quantstudio-6-7-pro-real-time-pcr-system.html
DeepLabCut v 2.3.3	Mathis et al. (2018)^34^	https://deeplabcut.github.io/DeepLabCut/README.html
SimBA v 1.70.2	Nilsson et al. (2020)^35^	https://github.com/sgold enlab/simba
nlme package v 3.1.163 in R	Pinheiro et al. (2026)^36^	https://doi.org/10.32614/CRAN.package.nlme
lme4 package v 1.1.37 in R	Bates et al. (2015)^37^	https://doi.org/10.32614/CRAN.package.lme4
emmeans package v 1.10.2	Lenth et al. (2026)^38^	https://doi.org/10.32614/CRAN.package.emmeans
mixOmics package v 6.26 in R	Rohart et al. (2017)^39^	https://mixomics.org/
DeSeq2 package v 1.42.1 in R	Love et al. (2014)^40^	https://bioconductor.org/packages/release/bioc/html/DESeq2.html
shinyGO 0.85	Ge et al. (2020)^41^	https://bioinformatics.sd state.edu/go/
BioRender	BioRender	https://www.biorender.c om/

### Experimental model and study participant details

Non sibling male CD-1 mice (ICR), aged 9–10 weeks old, were group-housed in individually ventilated cages (IVCs) with sufficient bedding and nesting material. Mice were bred in the animal facility of the Max Planck Institute of Biochemistry (Martinsried, Germany). Animals were maintained under a pathogen-free, temperature- and humidity-controlled environment (23°C ± 1°C and 55% ± 10%, respectively), on a 12-h light-dark cycle (lights on at 7 a.m.). Food and water were provided *ad libitum*. All experiments were performed in accordance with the European Communities’ Council Directive 2010/63/EU. All protocols were approved by the Ethics Committee for the Care and Use of Laboratory Animals of the government of Upper Bavaria, Germany and by the Institutional Animal Care and Use Committee (IACUC) of the Weizmann Institute of Science (Rehovot, Israel).

### Method details

#### Chronic social defeat stress paradigm

To preserve group integrity required for the social boxes, we implemented a modified version of the chronic social defeat stress (CSDS) paradigm. In this version, experimental mice were not co-housed with a dominant resident mouse after each defeat. Instead, they were returned to their original home cage with their regular group members. Maintaining group integrity is critical. If group housing is disrupted for more than 48 h, reintroducing mice into their original group can trigger heightened aggression, leading to serious fights, injuries, or even death. This constraint necessitated the modified protocol. To ensure sufficient stress induction despite the absence of post-defeat cohabitation, the duration of the protocol was extended from the standard 10 days to 21 consecutive days. Each day, experimental mice were introduced daily into the home cage of dominant resident mouse, triggering an attack on the intruder. Immediately upon presentation of the defeat posture by the subordinate mouse, mice were separated in order to avoid serious injuries. Experimental mice were then placed back in their home cages until the following day. Every day, for a total of 21 days, mice were defeated by a different CD1 aggressive mouse. Defeat encounters were randomized, with variations in starting time (between 8:00 a.m. and 6:00 p.m.) to decrease the predictability to the stressor and minimize habituation effects. Control mice were group-housed in the same room as the stressed mice during the CSDS protocol. Throughout the CSDS procedure, all home cages remained standard individually ventilated mouse cages (IVC cages). All animals were weighed 3 times a week, and their coat stated was assessed and scored on a scale of 1–4, according to the following criteria: [1] No wounds, well-groomed and bright coat, and clean eyes; [2] no wounds, less groomed and shiny coat, OR unclean eyes; [3] small wounds, AND/OR dull and dirty coat, and not clear eyes; [4] extensive wounds, OR broad piloerection, alopecia, or crusted eyes.

#### Experimental endpoint and tissue collection

At experimental endpoint, all mice received an isoflurane overdose and target tissues were dissected for molecular experiments. Trunk blood was collected for the assessment of basal CORT levels. Adrenal glands were dissected from fat and weighed. The brains were collected and snap-frozen using 2-methylbutane for dissection of regions of interest.

#### Plasma CORT measurement

Blood sampling was performed during endpoint (8:00 a.m.) by collecting trunk blood after decapitation. All samples were kept on ice until centrifugation (20°C), and 11 μL of plasma was removed for measurement of CORT. All plasma samples were stored at −20°C until CORT measurement took place. CORT concentrations were quantified by radioimmunoassay (RIA) using a CORT double antibody 125I RIA kit (sensitivity: 25 ng/mL; MP Biomedicals Inc.) following the manufacturer’s instructions. Radioactivity of the pellet was measured with a gamma counter (Wizard2 2470 Automatic gamma Counter; PerkinElmer). All samples were measured in duplicate, and the intra- and inter-assay coefficients of variation were both below 10%. Final CORT levels were derived from the standard curve.

#### The social box arena

The behavior of mice was assessed in ‘Social Box’ (SB) arenas, designed for automated tracking of individual and group behaviors, as described previously. The SB arena consists of a 60 cm × 60 cm box that includes the following objects: a closed nest, an open nest, 2 elevated ramps, an S-shaped wall, 2 water bottles, and 2 feeders. Food and water were available *ad libitum*. The arenas were illuminated at ca. 2 lx during the dark phase (12 h) and at ca. 200 lx during the light phase (12 h). A color-sensitive camera (Manta G-235C, Allied-Vision) was placed 1 m above the arena and recorded continuously. The fur of all mice was painted using four different hair dyes, in order to enable identification by automatic video tracking. Painting was carried out under mild isoflurane anesthesia (ca 10 min). Paint was applied using a paint brush, and excess color removed with tissues. Immediately after painting, mice were single-housed for approximately 2 h, before being regrouped in their original home cages. Mice were left to recover for several days before the start of the experiments. Mice trajectories were automatically tracked using the markerless pose estimation software DeepLabCut^34^^,^^42^^,^^43^ (DLC). Behavioral metrics were calculated by using a combination of custom-based software written in python, and Simple Behavior Analysis^35^ (SimBA). Following the completion of the CSDS (21 days), mice were transferred to the SB right before the onset of the dark phase and were kept there for 4 days (4 dark and 4 light phases: baseline). On the 4^th^ day, mice were temporarily removed from the SB and administered either (R,S)- ketamine (10 mg/kg) or saline intraperitoneally. Mice were subsequently returned to the SB, where they were monitored for the next 7 days (7 dark and 6 light phases). To avoid excessive habituation to the SB environment, each mouse group was swapped to a different SB (containing the bedding of a different group) every 2 days, following treatment administration. All animals within a group were always moved together. Exposure to another group’s environmental scent was intentionally used to limit habituation to the environment and to challenge ongoing social interactions, promoting exploration and social contact.

#### SB behavioral analysis

The following behaviors were automatically detected throughout the recordings: locomotion-related readouts (distance traveled, average speed, average acceleration, speeding, being motionless), interactions with objects in the SB (nest, open nest, two ramps, S wall, two feeders, two water sources) and social interactions (following, chasing, inter-mouse proximity, anogenital sniffing, head-to-head sniffing, head-to-side sniffing). For each behavioral readout, the total duration, number of events, and mean event duration were calculated across the time bins of interest. In addition, several secondary metrics were derived, including ramp preference, feeder preference, water-source preference, interaction with enrichment, sniffing-to-chase ratio, and chaser-to-chased ratio. All behavioral readouts were normalized to “time outside nest”, to account for different baseline exploration levels. Behavioral data were analyzed following a predefined, stepwise framework. First, data were analyzed across the full four-night post-stress period to capture overall group-level effects. In a second step, the first night was analyzed separately to assess the acute response to the novel environment, while nights 2–4 were analyzed together to examine more stable post-habituation behavioral patterns. Within this framework, nights 2 and 3 were emphasized because behavioral activity typically declines by night 4, reducing interpretability at later time points, consistent with previous Social Box studies.^26^^,^^29^^,^^44^^,^^45^ PCA analysis was performed in R using the mean values of these normalized behavioral readouts, summarized over windows of two, four, six or 12 h over the first four dark or light phases. These window sizes were systematically explored to characterize time-dependent differences between groups, and the ZT 14–18 interval was selected for the subsequent analysis because it provided the clearest separation between conditions. This temporal analysis was intentionally exploratory and designed to identify intervals of maximal behavioral divergence, rather than to test a pre-specified time window. Importantly, the PC scores were used as an exploratory and organizational tool, with the aim of summarizing many behavioral readouts and capturing underlying trends, such as relevant time windows and subgroup differences. Once these patterns were identified, we examined the underlying behavioral components driving the PCA effects. Linear mixed-effects models (LMMs) were fit in R using the “nlme” package,^36^ and generalized linear mixed-effects models (GLMMs) were fit using the “lme4” package.^37^ Type I ANOVA was performed. Pairwise comparisons were conducted using the “emmeans” package,^38^ and *p*-values were adjusted for multiple testing using the Benjamini–Hochberg false discovery rate (FDR) method. The sPLS-DA model was fit using the R package “MixOmics”.^39^

#### RNA extraction, quality control and bulk RNA-sequencing

Brain regions of interest (mPFC, vHipp) were dissected at the cryostat using a 1 mm punching needle, on 200 μm slices. Messenger RNA (mRNA) samples were extracted using the miRNeasy Micro Kit (Qiagen), according to the manufacturer’s instructions. RNA quality was assessed by Bioanalyzer, removing samples that showed RIN values < 8 and libraries prepared according to the “Illumina Stranded mRNA Prep” library preparation. Samples were sequenced at the National Genomic Infrastructure (Sweden) on NovaSeqXPlus (NovaSeqXSeries Control Software 1.2.0.28691) with a 151 nt(Read1)-10nt(Index1)-10nt(Index2)-151nt(Read2) setup using ‘10B’ mode flowcell. The Bcl to FastQ conversion was performed using bcl2fastq_v2.20.0.422 from the CASAVA software suite. The quality scale used was Sanger/phred33/Illumina 1.8+. The reference genome for annotation was GRCm38.

#### Differential expression analysis

Count data was imported in R package DeSeq2.^40^ Quality control led to removal of one outlier. Genes with low counts (total counts ≤10), and present in ≤3 samples were filtered out. The data was then analyzed with the formula Y ∼ SB + condition, to account for social group batch effects. Independent filtering was set to True during DEGs calculation, and genes were considered differentially expressed with padj<0.05. Because our primary objective was to investigate how ketamine and social hierarchy influence stress-related responses, we centered our analysis on group-level contrasts between stressed and non-stressed conditions, in line with the behavioral and physiological analyses.

#### GO enrichment analysis

Gene ontology (GO) enrichment analysis was performed using the web-based tool ShinyGo.^41^ Significantly DEGs were used as input, and enrichment was assessed across the Biological Process, Molecular Function, and Cellular Component GO databases. FDR-adjusted *p*-values were used to identify significantly enriched terms.

#### Reverse transcription and qRT-PCR

Total RNA was reverse-transcribed using the High-Capacity cDNA Reverse Transcription Kit (Thermo Fisher Scientific). Real-time PCR reactions were run in triplicate using the QuantiFast SYBR Green PCR Kit (Qiagen) and ABI QuantStudio6 Flex Real-Time PCR System and data was collected using the QuantStudio Real-Time PCR software (Applied Biosystems). Expression levels were calculated using the standard curve, absolute quantification method. The geometric mean of the endogenous expressed genes Rpl13 and *Gapdh* were used to normalize the data.

### Quantification and statistical analysis

For each graph, the sample size, center and dispersion measures as well as the statistical test used are described in the corresponding figure legend. When ANOVA was used, Shapiro-Wilk normality test was used to verify normality in data distribution. In case normality was not met, non-parametric test such as Kruskal-Wallis rank-sum test was used. When linear mixed-effects models were used, type I ANOVA was applied to assess the significance of fixed-effects Model assumptions, including normality of residuals and homoscedasticity, were verified using standard diagnostic plots (e.g., Q-Q plots, residual vs. fitted plots).
