# Supplementary material for: Social hierarchy shapes behavioral and transcriptional responses to chronic stress and ketamine in male mice
Source: iScience. 2026 Jul 21;29(8):116825. doi: 10.1016/j.isci.2026.116825 (PMC13392862; doi:10.1016/j.isci.2026.116825)
Supplement: Document S1. Figures S1–S6 [file mmc1.pdf]

iScience, Volume *29*

Supplemental information

Social hierarchy shapes behavioral and transcriptional responses to chronic stress and ketamine in male mice

Serena Gasperoni, Xiuqi Ji, Choham Sudre-Chinsky, Eduardo Cáceres Pajuelo, Fatemeh Sadat Zolfaghari, Otto Boldemann, Manar Manla Hasan, Tommaso Biagini, Daniil Umanski, Yair Shemesh, Aron Kos, Paula Fontanet, Alon Chen, and Juan Pablo Lopez

Supplementary Figures

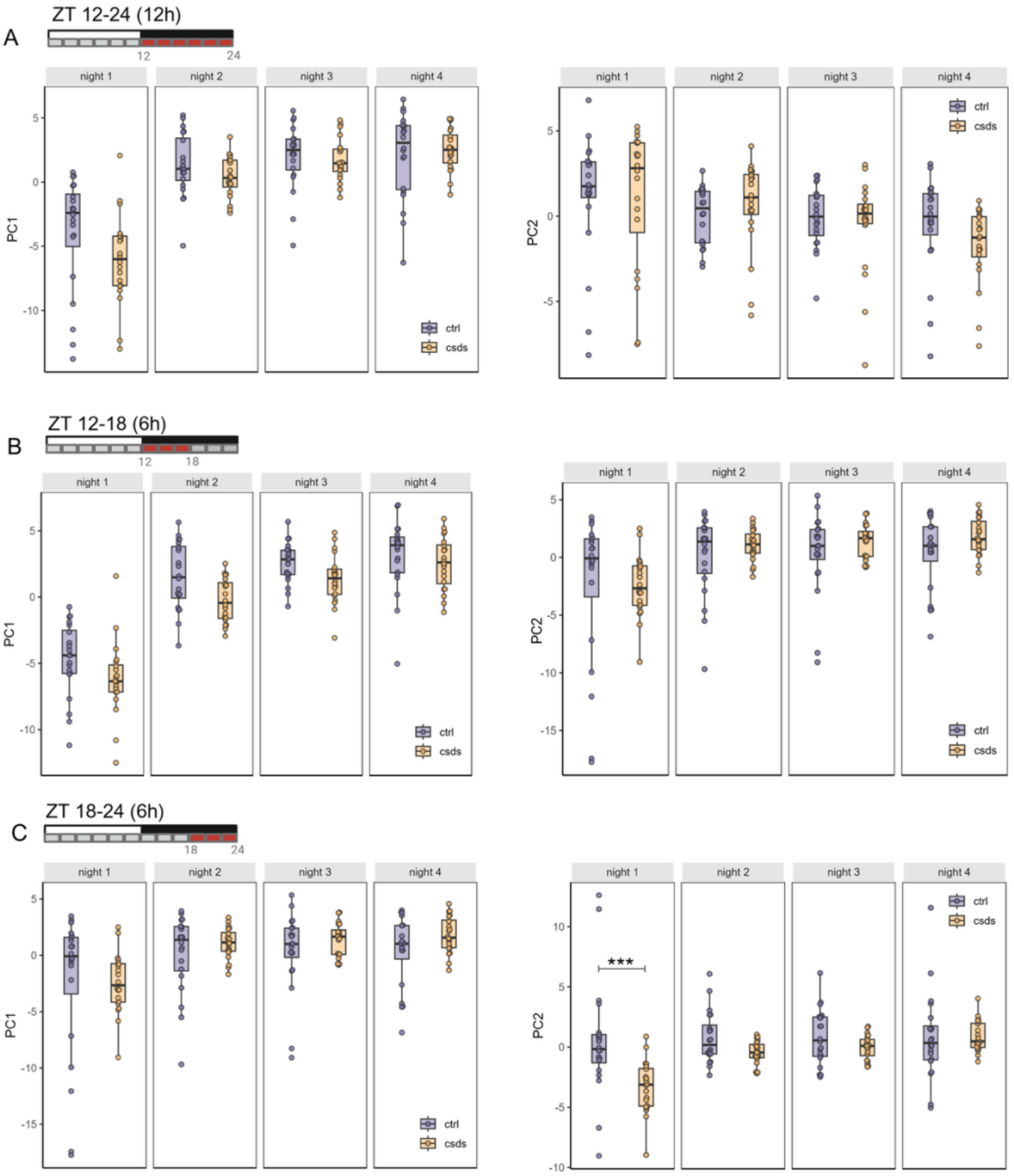

Figure S1. Evolution of PC1 and PC2 during the dark phase (12h and 6h intervals). Related to Figure 2. (A) Evolution of PC1 and PC2 in CTRL (violet) and CSDS (orange) mice at ZT12-124, separately for four days. **(B,C)** Evolution of PC1 and PC2 in CTRL (violet) and CSDS (orange) mice at ZT12-18 (B) and ZT18-24 (C), separately for four days. Data were analyzed using linear mixed-effect models with *group*, *day*, and their interaction as fixed effect, and *mouse ID* as a random intercept. Zeitgeber Time (ZT). *** $p < 0.001$.

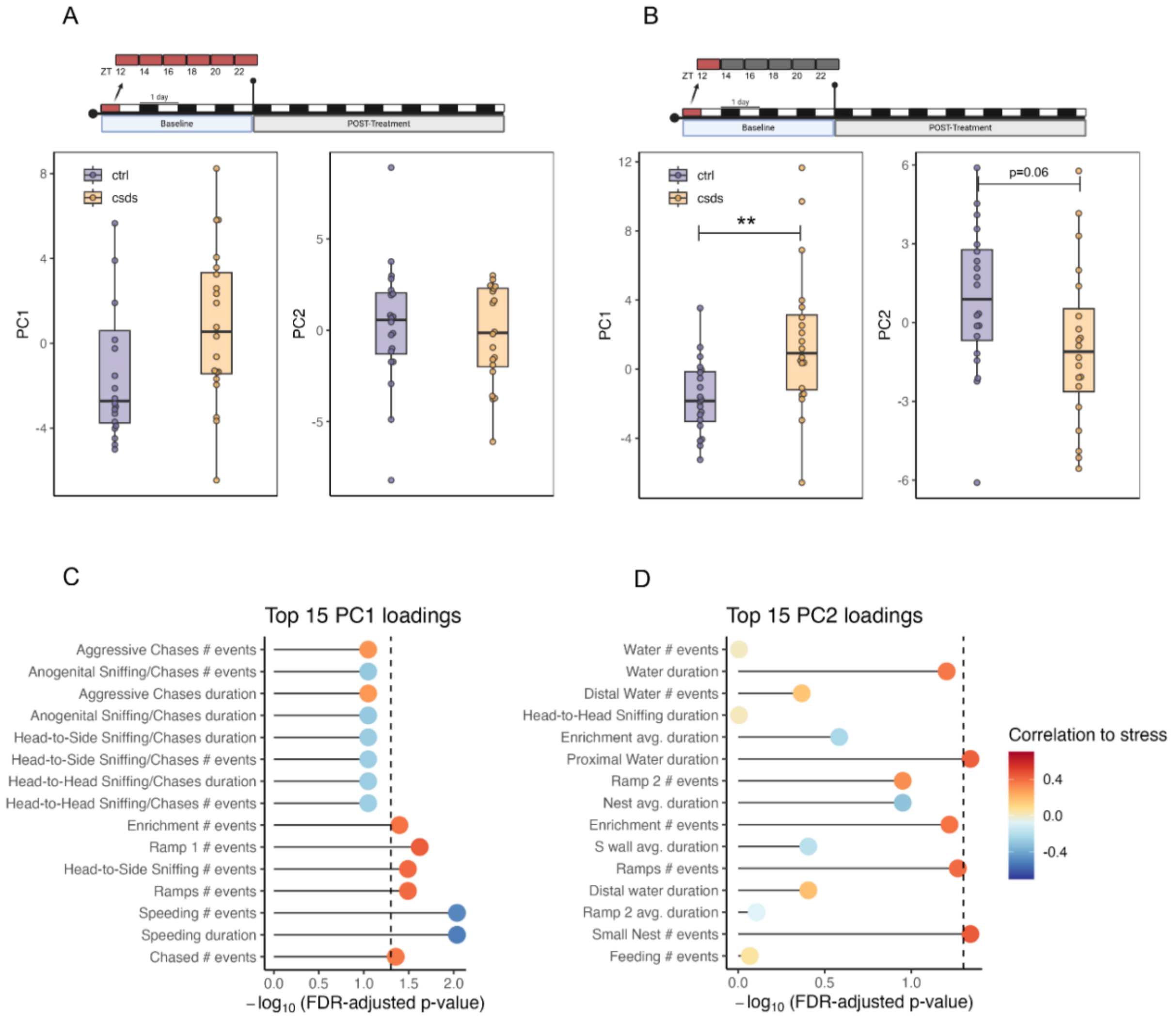

Figure S2. Acute effects of stress on SB exploration. Related to Figure 2. (A) PC1 and PC2 across groups on the first night (12h) in the SB. Data represents mean ± SEM; n=20 per condition. Unpaired *t* test, two-tailed. **(B)** PC1 and PC2 across groups on the first 2 hours in the SB. Data represents mean ± SEM; n=20 per condition. Unpaired *t* test, two-tailed. **(C,D)** Correlation (Pearson) of top 15 PC1 (D) and PC2 (E) loadings to group (CTRL, CSDS) membership, referring to the first 2 hours in the SB. Dashed line indicates -log (FDR-adj p-value) = 1.25; values on the right of the line indicate behavioral metrics significantly correlated with stress. Color represents direction and intensity of the correlation (using CTRL as reference). Zeitgeber Time (ZT) ** *p* <0.01.

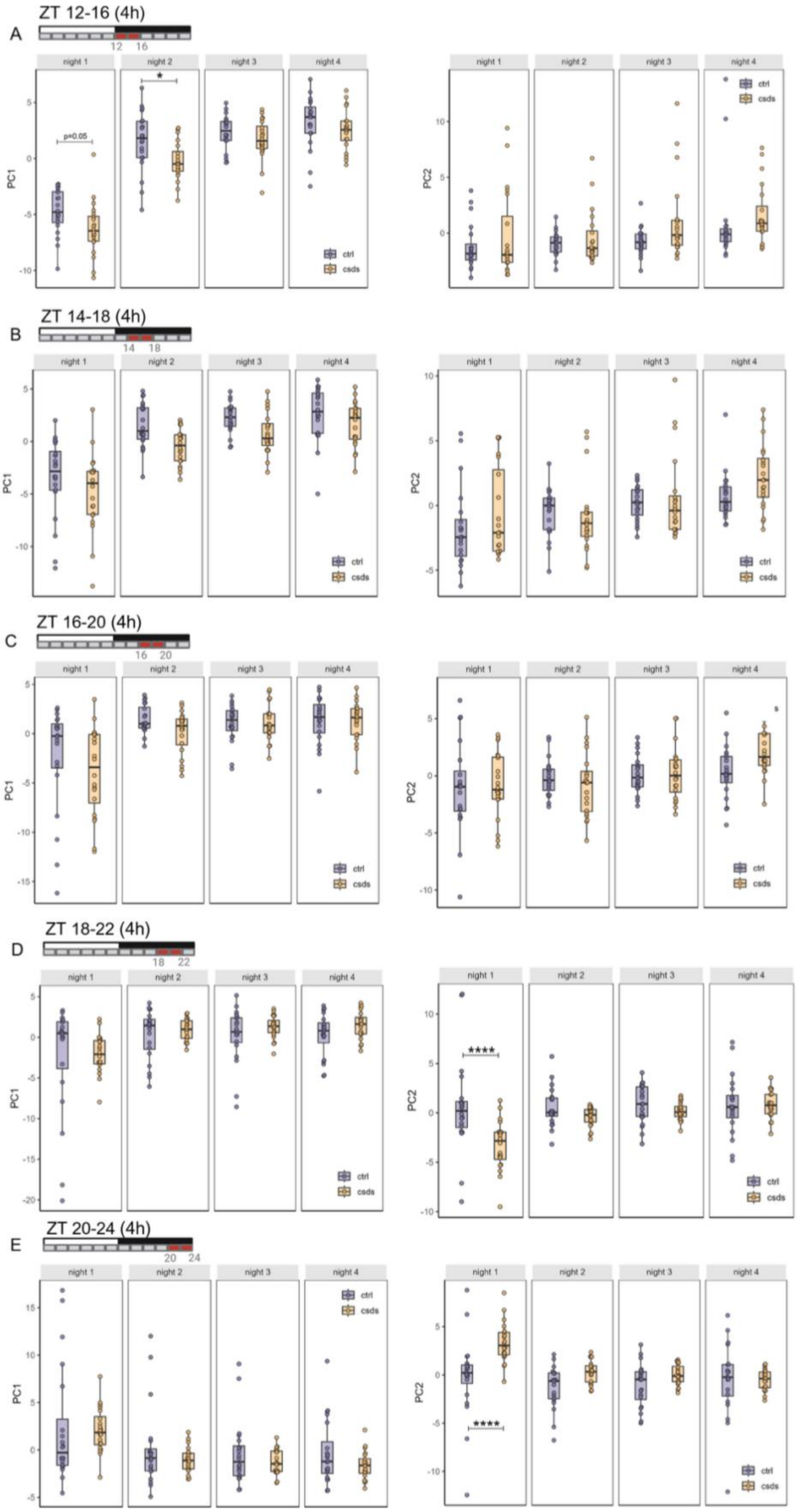

Figure S3. Evolution of PC1 and PC2 during the dark phase (4h intervals). Related to Figure 2. (A-E) Evolution of PC1 and PC2 in CTRL (violet) and CSDS (orange) mice at ZT12-16 (A), ZT14-18 (B), ZT16-20 (C), ZT18-22 (D) and ZT20-24 (E), separately for 4 days. Analyzed using linear mixed-effect models with *group*, *day*, and their interaction as fixed effect, and *mouse ID* as a random intercept. Zeitgeber Time (ZT). * p <0.05, **** p <0.0001.

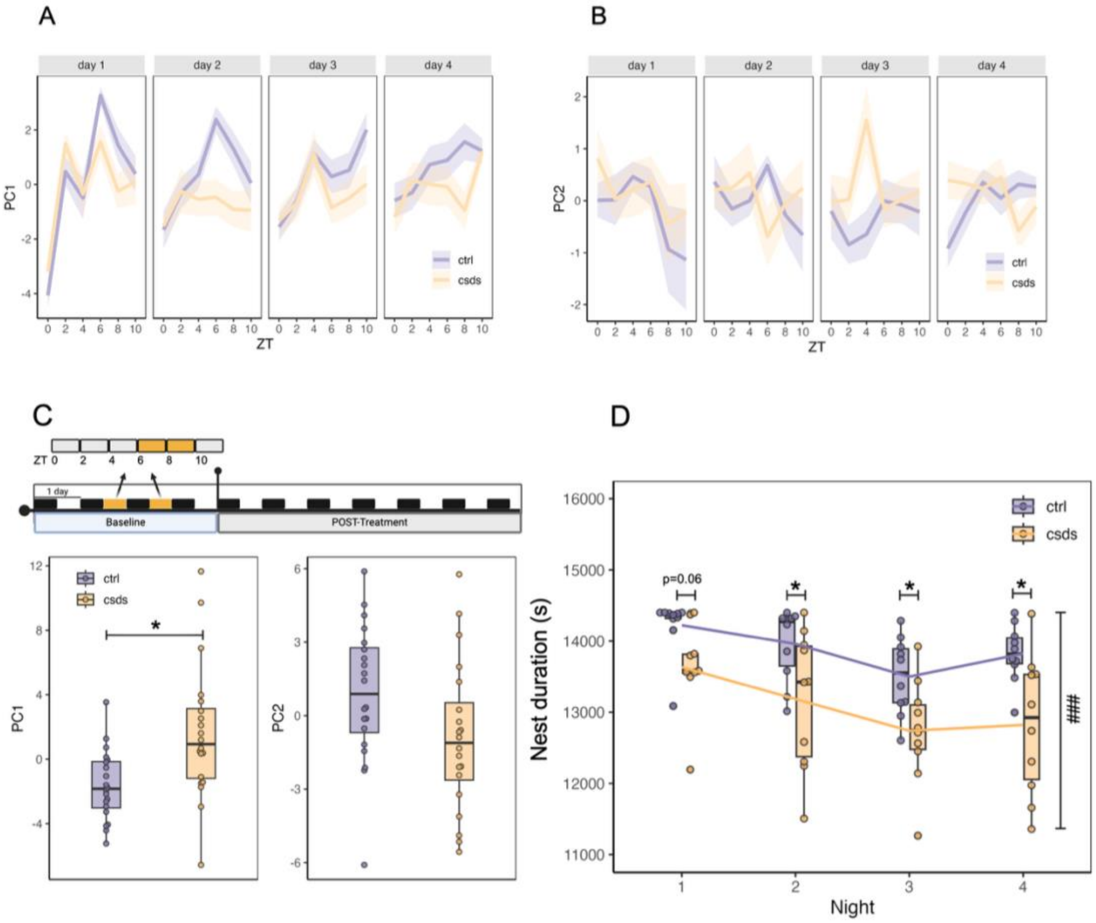

Figure S4. Evolution of PC1 and PC2 during the light phase. Related to Figure 2. (A,B) Evolution of PC1 (A) and PC2 (B) in CTRL (violet) and CSDS (orange) over 2-hours intervals, separately for four days. The x axis represents the Zeitgeber time (ZT), where ZT0 indicates the onset of the light phase. Data were analyzed using linear mixed-effect models with *group*, *day*, and their interaction as fixed effect, and *mouse ID* as a random intercept. **(C)** Selected time interval for dissecting the stable effects of stress on behavior during the light phase. Panels show PC1 and PC2 components across the groups. Data represents mean ± SEM; n=20 per condition. Data were analyzed using linear mixed-effect models with *group*, *day*, and their interaction as fixed effect, and *mouse ID* as a random intercept. **(D)** Evolution of time in the nest throughout the 4 days in the SB, at the selected time. Data were analyzed using linear mixed-effect models with *group*, *day*, and their interaction as fixed effect, and *mouse ID* as a random intercept. Zeitgeber Time (ZT) * p <0.05, ** p <0.01, ### p <0.001.

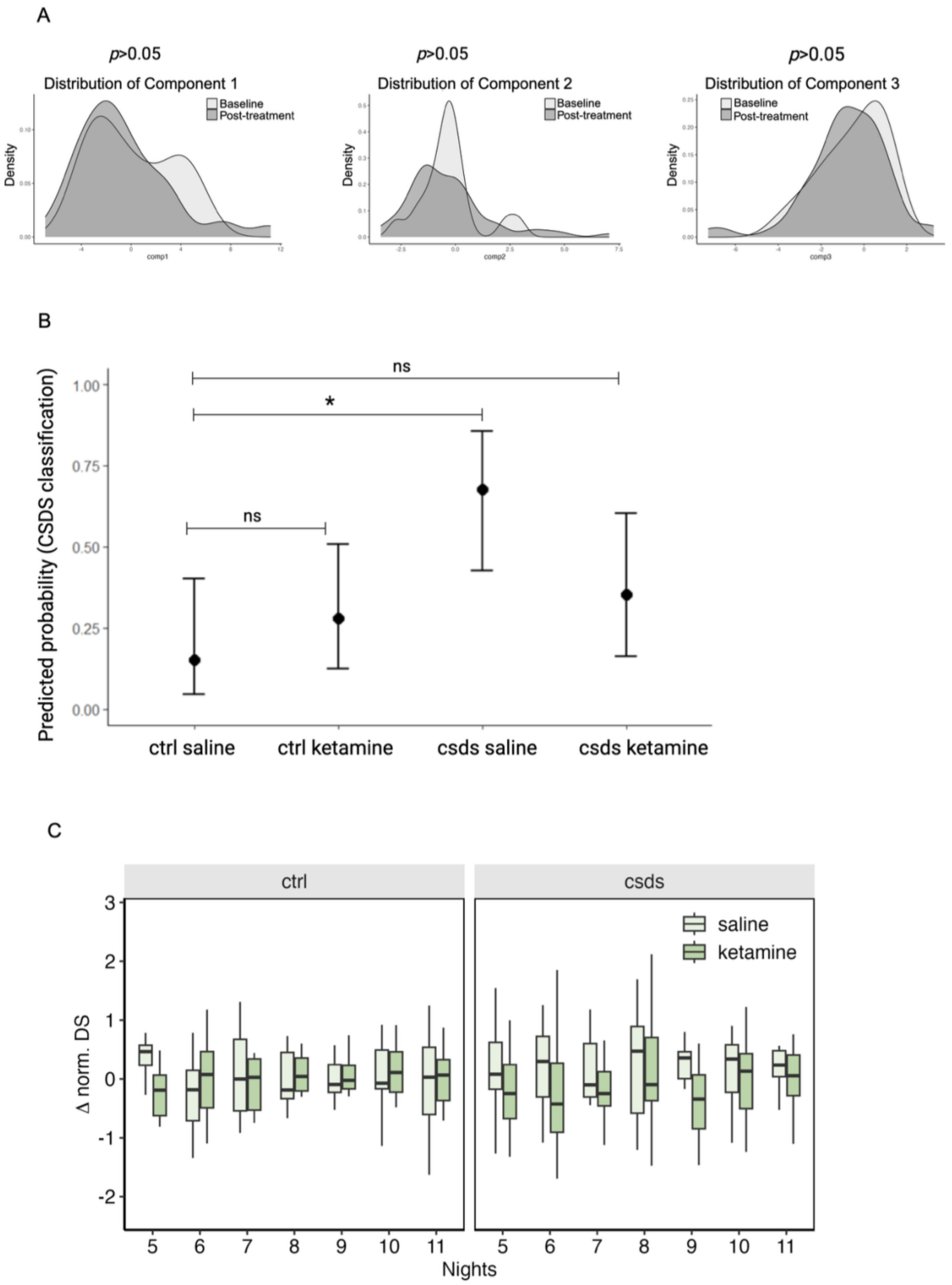

Figure S5. Treatment effects on sPLS-DA Components and normDS values. Related to Figure 5. (A) Comparison of distribution of baseline (light grey) and post-treatment (dark grey) sPLS-DA Component 1, 2 and 3, in saline-treated mice. Kolmogorov-Smirnov test. **(B)** Estimated marginal means (predicted probabilities) of CSDS classification for each group x treatment condition from a generalized linear mixed-effects model with group and treatment as fixed effects and subject ID as a random effect. Data refers to nights 1-7 following treatment administration. Error bars represent 95% confidence intervals. Post-hoc comparisons were FDR-adjusted. * $p < 0.05$**.** **(C)** Change (to baseline) in normDS values after treatment (*saline*, *ketamine*) in CTRL and CSDS mice. Data were analyzed using linear mixed-effect models with *group*, *treatment*, and their interaction as fixed effect, and *mouse ID* as a random intercept.

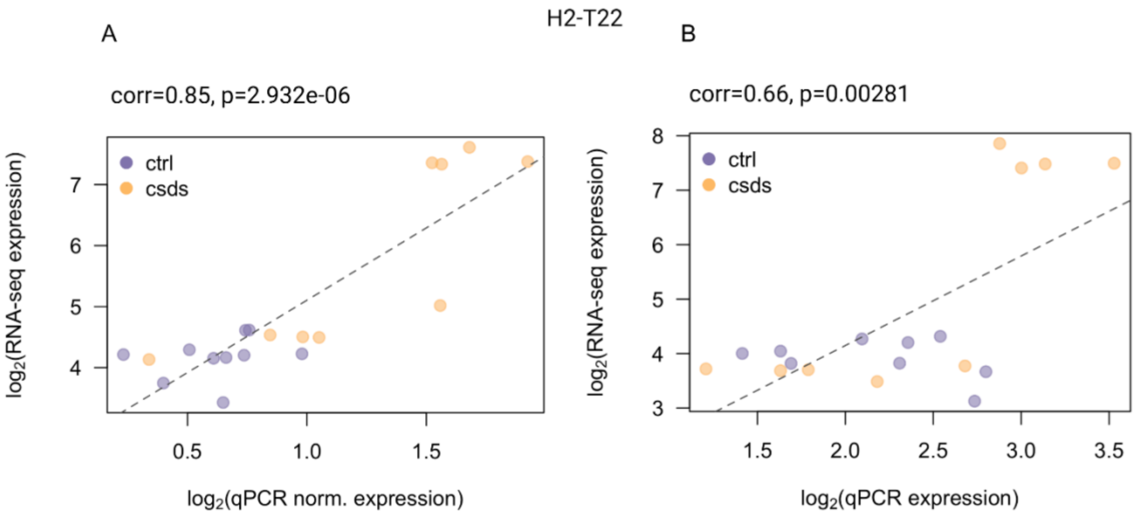

Figure S6. qPCR validation. Related to Figure 6. (A,B) Correlation (Pearson) of qPCR expression values of H2-T22 with bulk RNA-seq expression values in the vHipp (A) and mPFC (B).
